# Impact of Soil Cadmium on Land Snails: A Two-Stage Exposure Approach under Semi-Field Conditions Using Bioaccumulative and Conchological End-Points of Exposure

**DOI:** 10.1371/journal.pone.0116397

**Published:** 2015-03-19

**Authors:** Dragos V. Nica, Marioara Nicoleta Filimon, Despina-Maria Bordean, Monica Harmanescu, George Andrei Draghici, Simona Dragan, Iosif I. Gergen

**Affiliations:** 1 Faculty of Animal Sciences and Biotechnologies, Banat’s University of Agricultural Sciences and Veterinary Medicine “King Michael I of Romania” from Timisoara, Timisoara, Timis, Romania; 2 Faculty of Chemistry-Biology-Geography, West University of Timisoara, Timisoara, Timis, Romania; 3 Faculty of Food Processing Technology, Banat’s University of Agricultural Sciences and Veterinary Medicine “King Michael I of Romania” from Timisoara, Timisoara, Timis, Romania; 4 Faculty of Agriculture, Banat’s University of Agricultural Sciences and Veterinary Medicine “King Michael I of Romania” from Timisoara, Timisoara, Timis, Romania; 5 Department of Cardiology, “Victor Babes” University of Medicine and Pharmacy, Timisoara, Timis, Romania; Chinese Research Academy of Environmental Sciences, CHINA

## Abstract

Land snails are highly tolerant to cadmium exposure and are able to accumulate soil cadmium independently of food ingestion. However, little information exists on the kinetics of cadmium retention in terrestrial gastropods exposed to an increase in the soil cadmium content, over time. There is also little knowledge about how exposure to cadmium-polluted soils influences shell growth and architecture. In this context, we examined cadmium accumulation in the hepatopancreas and shell of juvenile *Cantareus aspersus* exposed to elevating high levels of cadmium in soil. Also, the toxicity of cadmium to snails was assessed using a range of conchological endpoints, including shell height, width, volume, allometry and integrity. Test snails, aged three months, were reared under semi-field conditions, fed an uncontaminated diet and exposed first, for a period of 30 days, to a series of soil cadmium concentrations, and then, for a second period of 30 days, to soils with higher cadmium content. Cadmium showed a dose-dependent accumulation in both the hepatopancreas and shell. The kinetics of cadmium retention in the hepatopancreas of snails previously exposed to cadmium-spiked soils was significantly influenced by a new exposure event. The shell was not a relevant bioaccumulator for soil cadmium. Under the present experimental conditions, only high cadmium exposure significantly affected either the shell growth or snail survival. There was no consistent effect on shell allometry, but the shell integrity, especially in rapidly growing parts, appeared to be affected by high cadmium exposure. Our results attest to the value of hepatopancreas for describing cadmium retention in land snails and to the difficulty of using conchological parameters in field surveys for estimating the environmental hazard of soil cadmium.

## Introduction

Cadmium (Cd) has attracted a lot of attention as a soil pollutant because of its persistence, toxicity and bioaccumulative potential along terrestrial trophic chains [1–3]. The United States Environmental Protection Agency (EPA) has classified Cd as a priority pollutant, whereas the European Community (EC) has included this metal on the Black List of chemicals [4]. Its average concentration in the earth’s crust ranges between 0.1 and 0.5 milligrams per kilogram dry weight (mg/Kg d. wt), with the highest values reaching up to 25 mg/Kg d. wt in sedimentary rocks [3]. However, human activities, especially mining, smelting, chemical industry, agriculture, and burning fossil fuels, account for most cadmium in anthropized soils [3,4]. Moreover, cadmium is more soluble and mobile in soils than other priority pollutant metals, e.g., mercury (Hg), zinc (Zn), chromium (Cr) or lead (Pb) [5]; therefore, its environmental occurrence at high levels represents a serious hazard for terrestrial ecosystems.

Among soil-dwelling invertebrates, land snails closely adhere to preconditions for serving as a pertinent bioaccumulator for soil cadmium [6–8]. These mollusks are able to sequestrate and detoxify Cd via complexation to specific cadmium metallothioneins [9], and hence can accumulate Cd far above environmental concentrations without showing any metabolic disorders [10]. *Cantareus aspersus* (syn. *Helix aspersa*) is the most often used terrestrial gastropod in environmental monitoring and assessment. This species has a well known biology and physiology [11] and is easily reared under both field and laboratory conditions [11,12]. It acquires Cd directly from soils [13], while showing a dose-dependent growth inhibition [13], and importantly, is able to access the nonlabile soil Cd pool [14]. This species is equally efficient as earthworms and much more sensitive than collembolas for assessing the degree of Cd-pollution in soils [8]. As a result, *C. aspersus* serves as an excellent study system for assessing the hazard of soil cadmium.

The kinetics of Cd transfer from soils to land snails has been extensively studied under both laboratory conditions [8,13–17] and semi-field conditions [18–20]. Cadmium was measured in the foot and viscera, not in the hepatopancreas although it accumulates primarily and in a dose-dependent manner in this organ [6,21]. The test specimens were continuously exposed for 2–12 weeks to constant Cd soil concentrations. A similar approach is used by the only standardized toxicity test with land snails for determining soil quality, viz. ISO-15952:2006 [22]. In anthropized areas, land snails are regularly exposed to successive soil contamination events, and hence to an increase over time in the soil Cd content. However, the aforementioned test does not consider this exposure scenario. Moreover, anthropized soils tend to exhibit elevated small-scale variability in metal concentrations [23,24]. Therefore, land snails after being exposed to a low level of metals can be exposed to elevating high levels of metals in soil in a short lifetime although they have very limited movement [25]. The kinetics of soil manganese retention in the hepatopancreas of snails previously exposed to Mn-spiked soils is significantly influenced by a new exposure event [26], and thus, we have hypothesized that a similar effect may occur in response to soil Cd exposure.

Most studies have used the fresh weight for assessing the influence of soil Cd on land snails [13,15,17,18], but rarely the shell growth, and only as a function of shell mass [16]. However, both these end-points of exposure are difficult to use under variable semi-field and field conditions. Thus, the snail weight depends on the state of hydration [27]. The shell mass closely relates to the shell thickness [28], and as a consequence, can vary in response to environmental factors, including metal pollution [29] and calcium bioavailability [30]. The increase in shell size is, by contrast, an irreversible biological process [31]; the shell volume may therefore serve as a more reliable test end-point for Cd exposure than either the fresh weight or shell mass. Moreover, Cd does accumulate in snail shells [32–36] and dietary Cd exposure was linked to changes in the shell volume/size [37]. The shell allometry, which reflects the covariation between different shell biometric traits [38], appears to be associated with long history of metal exposure in wild populations of land snails [1,34,39], but little information exists for short-term metal exposure (several months). The impact of Cd on the integrity of snail shells is currently not known despite the evidence for metal-induced abnormal shell growth in these mollusks [40] and shell damages in bivalve mollusks in response even to short-term Cd exposure [41].

At least two important questions arise from the above considerations: (1) Does a new exposure event change the kinetics of soil Cd retention in the hepatopancreas of land snails? (2) What is the relevance of the shell as an end point of exposure to Cd-contaminated soils? To test these hypotheses, we have implemented a two-stage experimental design, in which test snails are exposed to increasing cadmium levels in the soil during two successive 30-day exposure periods. Cadmium concentrations were measured in the hepatopancreas and shell, whereas the shell volume (estimated via the shell height and width) was used to assess the snail growth. The effect of soil Cd on shell allometry and integrity was also determined. Our results are important because they not only refine our knowledge on mechanisms underlying Cd accumulation in land snails, but also provide environmental scientists with an improved understanding on the relevance of snail shell as an end-point of metal exposure in (eco)toxicological field studies.

## Materials and Methods

### Ethical statement

The present study was performed on private land (45.8747° lat. N, 22.2117° long. E, 85 Temeresti, 305300 Faget, Romania) with the approval of the land owner (Stanila Doina). There were no specific permissions required to conduct the present experiments at this location. Ethical approval was not required for using *Cantareus aspersus* as study system in (eco)toxicological studies. However, our experiments were carried out in accordance with the internal guidelines of Banat’s University of Agricultural Sciences and Veterinary Medicine “King Michael I of Romania” from Timisoara (BUASVMT), Romania. These guidelines comply with the national and European recommendations regarding the protection and welfare of laboratory animals. The purchase of *C. aspersus* snails for this study did not involve endangered or protected species.

### Experimental design and snail rearing

The study was conducted outdoor in the village of Temeresti (Timis county, Romania). Six weeks before the start of the experiment (1st May 2011), 350 sub-adult *C. aspersus*, aged three months, were purchased from a snail farm in Romania (S.C. Edimpe Auto S.R.L., Muntenii de Sus, Vaslui county). The snails were maintained in 0.2 m^3^ polypropylene terrariums (0.50 x 1.00 x 0.40 m), in groups of 70 specimens per each terrarium. Each terrarium contained a 15 cm-deep layer of dark brown chernozem soil as a substrate (mean weight: 400 ± 14.32 g); the soil had a silty clay loam texture (clay: 27.5%, silt: 25.7%, sand: 46.8%), pH of 6.78, and 7.5% organic carbon content. The snails were fed ad libitum an uncontaminated fodder containing fodder chalk (15%), wheat meal (10%), corn meal (7%), soya grits (10%), corn germ grits (10%), sunflower grits (15%), monocalcic phosphate (3%), Inlavit (10%), wheat pollard (10%) and vitamin-mineral premix for piglets (10%) [26].

The terrariums were protected with a sun shade, thus providing environmental parameters that are similar to those encountered in the natural environment (in terms of temperature, photoperiod, sunlight, and air currents). The relative air humidity and soil moisture in the environment were checked twice a day (5.00 am and 23.00 pm, respectively) and their values within terrariums were adjusted accordingly by using a dew generator, a pressure sprayer and double distilled water. To avoid food contamination via soil contact, in each terrarium the fodder was placed in a 7-cm-diameter Petri dish. The daily activity schedule involved fresh fodder supply, monitoring snail fitness, removal of feces and uneaten food, and collecting the dead specimens. The terrariums were cleaned once a week with double distilled water.

Consistent with other studies, cadmium chloride (CdCl_2_) was used as a source for providing high Cd concentrations in the substrate, i.e., between 25 and 250 mg/Kg d. wt [13,15,16]. Such elevated soil Cd levels have been reported occur in areas adjacent to mines, smelters, battery plants, soldering and pigment producing plants; and in agricultural soils intensively amended with Cd-rich fertilizers, animal manures and sewage sludges [3]. The snails were exposed first, for a period of 30 days, to a series of soil Cd concentrations (i.e., the E1 phase), and then, for a second period of 30 days, to soils with higher Cd concentrations (i.e., the E2 phase). The rationality for applying a two-phase exposure setting was (1) to assess whether a new exposure event changes the Cd retention kinetics in land snails previously exposed to Cd-spiked soils (2) to improve the probability of observing the impact of Cd on snail shells by having different exposure regimes applied to juvenile specimens of different age and size. The duration of each phase was 30 days, which is consistent with the one advised by the ISO-15952:2006 standard, i.e., 28 days [22]. The spiking solutions had the following nominal concentrations (1) in the E1 phase: M1, control group; Cd1.1., 25 mg/L cadmium; Cd2.1., 50 mg/L cadmium; Cd3.1., 100 mg/L cadmium; Cd4.1, 125 mg/L cadmium; (2) in the E2 phase: M2, control group; Cd1.2., 50 mg/L cadmium; Cd2.2., 100 mg/L cadmium; Cd3.2., 200 mg/L cadmium; Cd4.2., 250 mg/L cadmium. The exposure route realistically simulated Cd transfer from soils to snails in natural environments, that is as a cumulative process that occurs via mixed dermal (diffusion through the foot epithelium) and digestive routes (ingestion of soil particles and detritus) [16,18,20]. For each experimental phase, Cd toxicity was assessed under static exposure conditions (substrate without renewal).

The substrate was sieved before being spiked by using a 5-mm mesh sieve to remove coarse materials (e.g., roots, rocks, macro-organic matter) and particles larger than 5 mm. Cadmium chloride (CdCl_2_, 99.99% trace metal basis, Sigma-Aldrich Chemie GmbH, Buchs, Switzerland) was used to prepare the corresponding spiking solutions. After spiking, the substrate was homogenized in batches in a Waring blender (about 1,200 g per each Cd treatment). The substrate followed an equilibration period for one week before the start of each experimental phase. The soil pH was measured at 0, 30 and 60 days of exposure by using a digital pH meter.

At the beginning of the E1 phase (15th June 2011) the snails were sorted based on their size. The shell volume was estimated as a function of shell height (SH) and shell width (SW). These conchological features were measured SW to the nearest 0.01 mm with a digital caliper; the protocol was compiled from the malacological literature [28]. Finally, 255 snails of similar shell height and width were selected and split in five samples (51 snails per bulk sample), one not exposed (the control group) and four exposed to different soil Cd concentrations. Three replicate jars (of 17 *C. aspersus* juveniles each) were used per each Cd treatment. All snails were numbered with black water-insoluble paint, thus allowing the precise identification of each specimen.

Topsoil samples (five samples of 5 g each) were collected for each replicate jar one day after the start of the E1 phase (day 0). The samples were dried (22°C, 7 d), disaggregated, homogenized, sieved to 2 mm and then stored in self-sealing sterile paper pouches for further analysis. The daily activity schedule was similar to the one followed during the pre-exposure period. Whenever there was the case, the snails were detached from the terrarium walls were to prevent their entrance into estivation. The pattern of shell damage in response to Cd exposure was also determined in snails that were alive at the end of this phase of exposure.

For each replicate jar, five specimens were fasted for 48 h at the end of the E1 phase (day 30) and then sacrificed by freezing (−20°C, 4 h). After thawing, the soft body was removed from the shell by using a hemostat. Among soft tissues, only the hepatopancreas (syn. digestive glands or midgut glands) was considered in the present work. The samples were washed in double distilled water and then stored until processing in the lab (chest freezer at −20°C). The shells were also sampled; prior to chemical analysis, they were rinsed three times with sterile double distilled water, dried with sterile paper towels and then gently crushed in a mortar. The same experimental protocol was followed for the E2 phase (which started on 15th July 2011).

### Chemical analysis

The concentrations of Cd in samples (soil, snail hepatopancreas and shell) were determined in the Environmental Research Test Laboratory (Banat’s University of Agricultural Sciences and Veterinary Medicine “King Michael I of Romania” from Timisoara, Romania). The soil samples were weighed on an analytical balance to the nearest 0.01 mg. Cadmium was extracted from soils to solution by wet extraction with nitric acid (HNO_3_). To this end, the soil samples were digested in 50 ml of 0.5 N HNO_3_ at a ratio of 1:10 soil and nitric acid solution (22°C, 24 h) and then filtered through ash-free filter paper. For each sample, 10 ml of filtrate were transferred in a sterile polyethylene tube and brought up to 45 ml with 35 ml of 0.5 N HNO_3_. Various acid mixtures (e.g., containing HClO_4_, HCl, HNO_3_ and/or HF) provide a higher percentage extractability for soil Cd [42], but wet digestion with HNO_3_ is a convenient, simple and cost-effective alternative for serial measurements. Metal mobility is key when evaluating the environmental risk of anthropized soils [42]. Using a 1:10 soil-to-HNO_3_ ratio for wet extraction allows a reliable determination of mobile metals from highly polluted soils [43], as is the case of our study.

The hepatopancreas samples were thawed, oven dried (105°C, 24 h), weighed to the nearest 0.01 mg by using an analytical balance and then calcinated in a muffle furnace (550°C, 4 h). The ash was dissolved in 10 ml of 0.5 N HNO_3_ and filtered through ash-free filter paper. The volume was brought to 45 ml with 35 ml of 0.5 N HNO_3_. After being calcinated (450°C, 48 h) to reduce the shell organic matter content, the shells were digested in 10 ml of HNO_3_ suprapure (Merck, 65% suprapure), and brought to 45 ml with 35 ml of double distilled water.

The concentrations of Cd in the filtrates were assessed by flame atomic absorption spectrophotometry (FAAS) with high resolution continuum source (Model ContrAA 300, Analytik Jena, Germany). All results were expressed as milligram per kilogram dry weight (mg/kg d. wt). Mix standard solutions (1000 mg/L) of Cd- ICP multielement standard solution IV CertiPUR were purchased from Merck. Double distilled water (spectroscopic pure) was used to prepare the reagents and standard solutions. All glassware was treated with Pierce solution 20% (v/v), rinsed with cold tap water, washed with 20% (v/v) nitric acid and then rinsed again with double distilled water. For quality control purposes, all blanks and triplicate samples were analyzed during the procedure. NCS Certified Reference Material-DC 85104a and 85105a (China National Analysis Center for Iron&Steel) was analyzed for quality assurance. The percent recovery mean was 105%. The variation coefficient was 6%. Cadmium detection limit, as measured by using the calibration curve, was 0.01 mg/Kg d. wt. The blank reagent and standard reference soil were included into each sample batch to verify the accuracy and precision of the digestion procedure, as well as for the subsequent analyses.

The content of other trace metals (e.g., Cu, Mn) in snail and soil samples was also determined. The measured values and the potential interference of metal-metal interactions in Cd accumulation in land snails will be presented in a future study.

### Statistical analysis

Normality and homogeneity of variance for Cd concentrations in the soil, the snail hepatopancreas and shell were verified by using an Anderson-Darling test and a Bartlet’s test, respectively. A simple linear regression analysis was applied to Cd concentrations in spiking solutions and substrates to check the accuracy of the spiking procedures. Differences in cadmium content of hepatopancreas and shell in each experimental phase were analyzed using a one-way ANOVA. Linear regressions were then conducted to investigate the relationships between Cd concentrations in the soil and those measured in either the hepatopancreas or the shell. To this end, five alive snails (per each replicate jar) were randomly selected and sacrificed at the end of each exposure phase. Regression slopes that were significant during both experimental phases resulted in the use of a one-way covariance analysis (ANCOVA) to distinguish between the subsequent kinetics of Cd accumulation in the hepatopancreas and/or shell. If significant differences were observed, another regression analysis was performed to examine the effect of a new exposure event on the kinetics of Cd accumulation in snails previously exposed to Cd-spiked soils (excluding the control snails). The difference in soil Cd levels between the E2 and E1 phase was used as a predictor variable, whereas the corresponding differences for the hepatopancreas/shell were considered as criterion variables.

The shell robustness, expressed as the relative shell height (RSH) via the ratio between SH and SW [1,39], was used to assess the allometry of the growing shells. All conchological parameters (SH, SW, RSH) were checked for normality (using an Anderson-Darling test) and for the homogeneity of their variance (using a Bartlet’s test). An ANOVA test was further run to assess differences in growth in SH and SW after different times of exposure (day 0, 30, and 60), with a post hoc Dunnet’s test being applied for significant differences among treatments. A similar approach was used for RSH. Finally, linear regressions were applied to assess the relationships between Cd addition to the shell, as a predictor variable, and either SH or SW as criterion variables. The control snails were excluded from this statistical analysis.

At the end of each experimental phase, a Chi-Square test was conducted to determine the differences in frequency of damaged shells, with emphasis on damages occurring at the shell growing edge (i.e., around the aperture). Survival analysis was then carried out for each treatment by using a Log-Rank test, followed by application of Breslow’s tests for post hoc analysis. Survival rates of control groups during the E1 and E2 phase were compared using a Chi-Square test to assess the contribution of environmental factors to snail mortalities. The proportion of the snails surviving at successive times for each treatment was assessed using a Kaplan-Meier curve. The treatment was considered complete for the dead specimens and censored for the snails who were still alive at the end of the study. The average survival time was determined as the area under the Kaplan-Meier estimate of the survival curve [44].

## Results

The soil pH remained relatively constant throughout the study (pH = 6.72–6.78). At the end of the experiment, the investigated snails were still sub-adults. The snails were generally observed spending 2–3 hours while feeding on the fodder in the Petri dishes before detaching from the substrate. Although the consumption rates were not measured, a small proportion of snails was observed in the E2 phase to almost completely reject the fodder; about 7.5% of the Cd4.2. snails and 4.25% of the Cd3.2. snails, respectively.

The concentrations of Cd in control soils were below the detection limit (0.01 mg/Kg d. wt) at the start of both experimental phases (Table 1). Regression analysis showed that the soil spiking was properly conducted, since the soil Cd level increased with the nominal concentration of Cd-spiking solutions (*p* = 0.000, *R* ^*2*^ = 0.992). The fodder Cd content also fell below the detection limit of FAAS. Therefore, any deposition of cadmium in the hepatopancreas was independent of food ingestion and resulted from its direct transfer from soils to snails.

**Table 1:** Mean (and SD) for concentrations of Cd in the soil and the snail hepatopancreas and shell.

	Cd concentrations (mg/Kg d. wt)						
	Day 30				Day 60		
Cd treatment	Soil	Hepatopancreas	Shell	Mn treatment	Soil	Hepatopancreas	Shell
M1	< 0.01	< 0.01	< 0.01	M2	< 0.01	< 0.01	< 0.01
Cd1.1.	24.38 (0.50)	< 0.01	< 0.01	Cd1.2.	48.50 (1.20)	43.42 (7.11)	1.87 (0.69)
Cd2.1.	48.52 (1.14)	< 0.01	< 0.01	Cd2.2.	97.42 (1.62)	128.02 (8.82)	7.13 (1.15)
Cd3.1.	95.88 (2.78)	56.72 (6.59)	2.68 (0.93)	Cd3.2.	194.97 (1.79)	421.97 (15.15)	4.04 (1.07)
Cd4.1.	121.81 (1.66)	180.72 (16.28)	5.35 (1.18)	Cd4.2.	243.72 (1.62)	482.53 (14.56)	9.22 (1.80)

The values were measured at the end of the E1 phase (day 30), and the end of the E2 phase (day 60).

### Kinetics of cadmium in hepatopancreas

The control groups showed no detectable accumulation of Cd in the hepatopancreas (Table 1), as well as the Cd1.1. and Cd 1.2. snails. The hepatopancreas had up to eightfold higher concentrations of Cd in the E2 phase than in the E1 phase, but the measured values were not at equilibrium. The hepatopancreas Cd content increased with soil Cd concentrations, irrespective of experimental phase (ANOVA, E1 phase: *p* = 0.000; E2 phase: *p* = 0.000).

**Fig 1:**
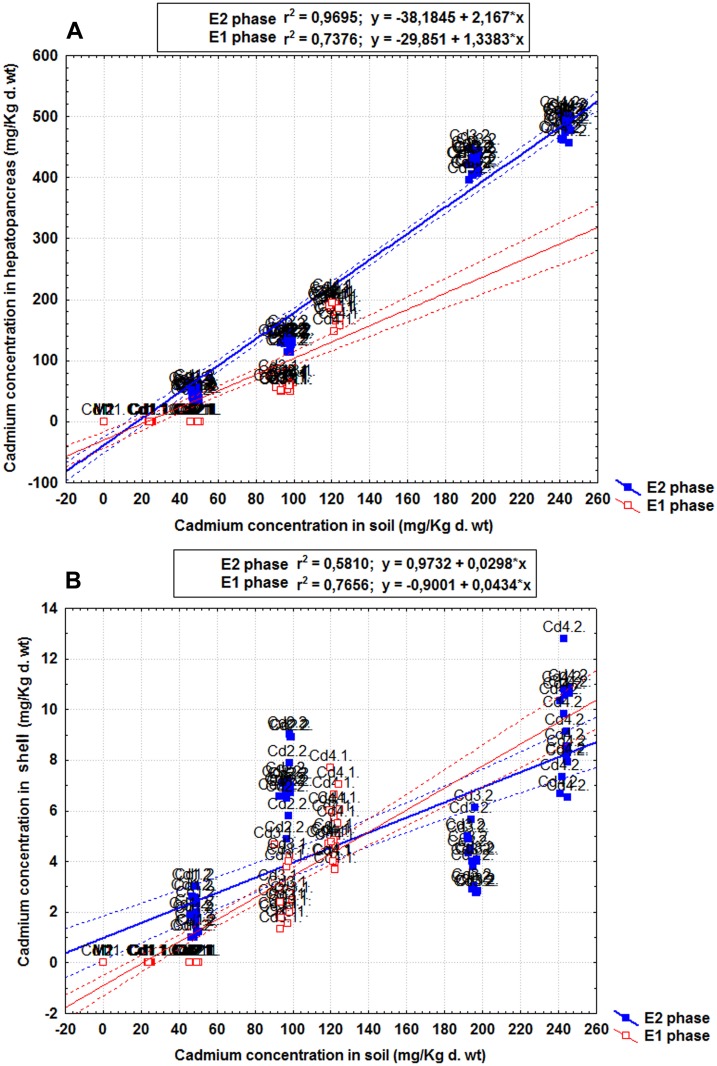
The soil-to-organ regressions in the E1 and E2 phases. A) snail hepatopancreas; B) snail shell. The scatter plots correspond to the mean cadmium concentrations in the soil, the snail hepatopancreas and shell.

The soil-to hepatopancreas regressions produced a very good fit to the data both in the E1 phase (*p* = 0.000, *R* ^*2*^ = 0.738) and E2 phase (*p* = 0.000, *R* ^*2*^ = 0.969), but the corresponding slopes (Fig. 1A) were significantly different one from each other (ANCOVA, *p* = 0.000). Moreover, the increase in soil Cd content resulting from a new exposure event was a very good predictor for Cd addition to the hepatopancreas of snails previously exposed to Cd-spiked soils (*p* = 0.000, *R* ^*2*^ = 0.920).

### Kinetics of cadmium in shell

Cadmium accumulated to much lower levels in the shell than in the hepatopancreas. The measured values generally increased with exposure time and dose, indicating that Cd did not reach equilibrium in the snail shell (Table 1). The shell of control snails, Cd1.1 snails and Cd2.1. snails revealed no detectable accumulation of Cd. Within the same group, the shell Cd content was always higher in the E2 phase than in the E1 phase. Analysis of variance showed that cadmium accumulations in the shell differed significantly among treatments during each exposure phase (ANOVA, E1 phase: *p* = 0.000; E2 phase: *p* = 0.000). The soil-to-shell regressions (Fig. 1B) were highly significant, but their coefficients of determination were lower than those observed for the corresponding soil-to-hepatopancreas regressions (E1 phase: *p* = 0.000, *R* ^*2*^ = 0.737; E2 phase: *p* = 0.000, *R* ^*2*^ = 0.581). Comparison of their regression slopes (Fig. 1B) revealed different kinetics of Cd retention in the shell between the E1 and E2 phase (ANCOVA, *p* = 0.000), but the effect of a new exposure event was not significant (*p* = 0.390, *R* ^*2*^ = 0.112). Therefore, the dynamics of Cd accumulation in the shell appears to be related not only to soil Cd concentrations, but also to other contributing factors.

### Cadmium toxicity

Globally speaking, the shell height varied little among test snails after 0, 30, and 60 days of exposure to Cd-spiked soils (Table 2). Similar results were obtained for the shell width (Table 3), as well as for the relative shell height (Table 4). At the start of the experiment (day 0), the investigated conchological traits were highly homogeneous (ANOVA, SH: *p* = 0.234; SW: *p* = 0.244; RSH: *p* = 0.475). No significant growth inhibition or allometric changes were found after 30 days of Cd exposure (ANOVA, SH: *p* = 0.525; SW: *p* = 0.243; RSH: *p* = 0.373). After 60 days, soil Cd did not influence the shell allometry (ANOVA, *p* = 0.324), but reduced the growth in shell volume (ANOVA, SH: *p* = 0.042; SW: *p* = 0.045). Significant decrease in shell height and shell width when compared to the control occurred in the Cd3.2. snails (Dunnet’s test, SH: *p* = 0.043; SW: 0.027) and the Cd 4.2. snails (Dunnet’s test, SH: *p* = 0.039; SW: *p* = 0.045), but not for the snails exposed to lower soil Cd levels (Dunnet’s test, *p* ≥ 0.211). However, no relationships existed between the rate to which Cd incorporates in the shell and growth in both SH (*p* = 0.497, *R* ^*2*^ = 0.126) and SW (*p* = 0.206; *R* ^*2*^ = 0.367).

**Table 2:** Mean (and SD) for SH in snails exposed to Cd-spiked soils.

	Mean height (cm)			
Day 0	Cd treatment	Day 30	Cd treatment	Day 60
1.91 (0.23)	M1	1.98 (0.27)	M2	2.04 (0.28)
1.84 (0.16)	Cd1.1.	1.87 (0.15)	Cd1.2.	1.92 (0.15)
1.90 (0.17)	Cd2.1.	1.96 (0.18)	Cd2.2.	2.07 (0.16)
1.83 (0.18)	Cd3.1.	1.85 (0.23)	Cd3.2.	1.85 (0.17)*
1.84 (0.14)	Cd4.1.	1.87 (0.16)	Cd4.2.	1.89 (0.14)*

Marked boxes (*) indicate significant differences as compared to the reference group (Dunnet’s test, p < 0.05).

**Table 3:** Mean (and SD) for SW in snails exposed to Cd-spiked soils.

	Mean width (cm)			
Day 0	Cd treatment	Day 30	Cd treatment	Day 60
2.03 (0.22)	M1	2.13 (0.28)	M2	2.17 (0.24)
1.96 (0.19)	Cd1.1.	1.99 (0.19)	Cd1.2.	2.05 (0.18)
2.01 (0.14)	Cd2.1.	2.09 (0.16)	Cd2.2.	2. 12 (0.15)
1.92 (0.21)	Cd3.1.	1.95 (0.25)	Cd3.2.	1.98 (0.20)*
1.94 (0.13)	Cd4.1.	1.95 (0.12)	Cd4.2.	1.97 (0.12)*

Marked boxes (*) indicate significant differences as compared to the reference group (Dunnet’s test, p < 0.05).

**Table 4:** Mean (and SD) for RSH in snails exposed to Cd-spiked soils.

	Mean RSH			
Day 0	Cd treatment	Day 30	Cd treatment	Day 60
0.94 (0.03)	M1	0.94 (0.02)	M2	0.93 (0.03)
0.93 (0.02)	Cd1.1.	0.93 (0.02)	Cd1.2.	0.93 (0.02)
0.94 (0.04)	Cd2.1.	0.94 (0.05)	Cd2.2.	0.94 (0.04)
0.94 (0.02)	Cd3.1.	0.94 (0.02)	Cd3.2.	0.93 (0.03)
0.94 (0.03)	Cd4.1.	0.94 (0.03)	Cd4.2.	0.94 (0.03)

**Fig 2:**
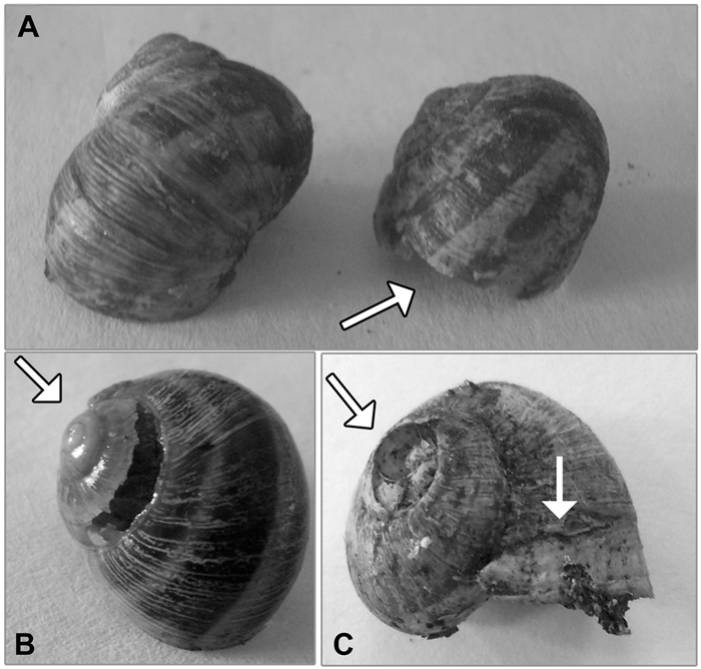
Photograph of different types of shell damages (indicated with arrows) in *C. aspersus* sub-adults. A) Irregular edges around the aperture; B) Shell erosion along the suture line; C) Thinned, fragile, and irregular edges around the aperture and broken apex.

Macroscopic examination of the shells of snails alive at the end of each exposure phase revealed different types of shell damages. The pattern of damage consisted mainly of thinned, fragile, and irregular edges of the shell at the growing edges (around the aperture), and, more rarely, deep wormholes in the body whorl and shell erosion along the suture line (Fig. 2). Similar shell deformations were observed in the snails who died during the experimental period. The frequency of snails with damaged shells tended to increase with exposure duration and dose, and was above 30% after 60 days of exposure to the highest Cd treatments (Table 5). For the incidence of damaged shells, significant differences existed among treatments in the E2 phase, but not in the E1 phase (Chi-Square test, E1 phase: *p* = 0.392; E2 phase: *p* = 0. 027).

**Table 5:** Snail survival in percentage and mean survival time (and SD) for each Cd treatment.

	Day 30				Day 60		
Cd treatment	Survival (%)	Mean survival time (days)	Shell damage (%)	Cd treatment	Survival (%)	Mean survival time (days)	Shell damage (%)
M1	96%	29.22 (3.66)	12%	M2	80%	27.65 (1.13)	8.25%
Cd1.1.	98%	29.86 (0.98)	10%	Cd1.2.	81.25%	28.64 (4.13)	12.13%
Cd2.1.	98%	29.86 (0.97)	10%	Cd2.2.	73.08%	26.04 (7.75)	3.57%
Cd3.1.	92%	29.42 (0.34)	14%	Cd3.2.	50%	25.07 (6.98) *	30.77%
Cd4.1.	84%	28.45 (4.60)	18%	Cd4.2.	58.33%	21.73 (11.16) *	41.67%

Marked boxes (*) indicate significant differences as compared to the reference group (Breslow’s test, p < 0.05).

**Fig 3:**
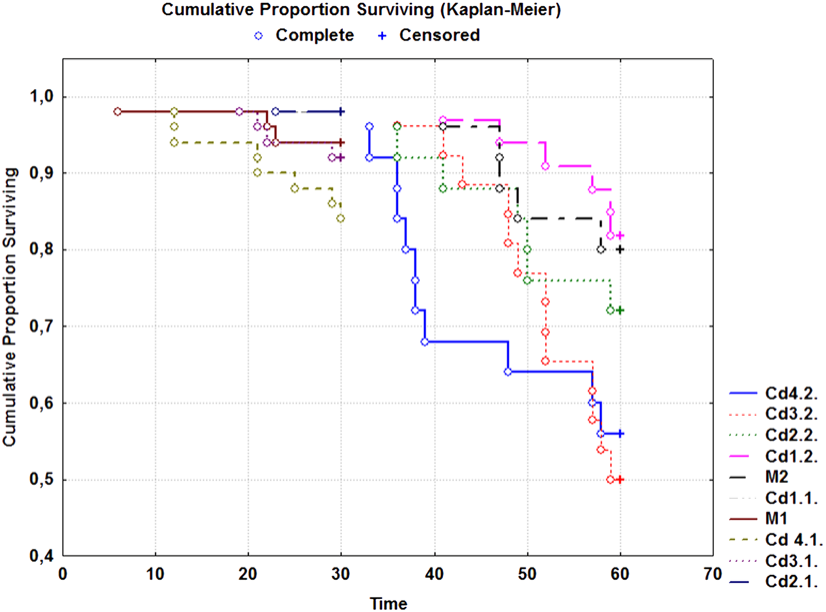
Kaplan-Meier survival curves in the E1 phase (left) and E2 phase (right). The complete data relate to the death snails, whereas the censored data are associated with the living individuals.

In the E1 phase, the survival rates (Table 5) were similar among different treatments and there was no clear pattern in the temporal distribution of snail deaths (Fig. 3). In the E2 phase, by contrast, the mortality rates were higher in the Cd-exposed snails than those observed in the control snails, excepting the lowest treatment (Table 5). Mortalities during the E2 phase reached a peak between 30 and 40 days of exposure for the Cd4.1. snails; and between 42 and 52 days of exposure for the Cd4.2. snails (Fig. 2). Survival analysis showed significant differences in mortality distribution across groups in the E2 phase, but not in the E1 phase (Log-Rank test, E1 phase: *p* = 0.076; E2 phase: *p* = 0.022). Post hoc testing revealed that, for the two highest treatments, soil cadmium contributed significantly to snail mortalities (Breslow’s test, *p* ≤ 0.048), whereas for the other treatments of the E2 phase, the snails showed no significant differences in survival dynamics as compared to the reference group (Breslow’s test, *p* ≥ 0.513). Moreover, the overall survival rates in control groups differed significantly between the E1 and E2 phase (Chi-Square test, *p* = 0. 001).

## Discussion

### Kinetics of cadmium in hepatopancreas

To our knowledge, this is the first study to take into account an exposure scenario reflecting an increase over time in soil Cd concentrations and to provide quantitative data on Cd direct transfer from soils to the hepatopancreas of land snails. Cadmium did not accumulate in the hepatopancreas after 30 days of exposure to soil Cd concentrations of up to 50 mg/Kg d. wt. Soil alkalinization and organic matter content decrease Cd bioavailability to land snails [17]. Therefore, we suggest that Cd bioavailability in the soil was low, thus limiting its uptake by land snails. Exposure above 50 mg/Kg d.wt in the soil resulted in high Cd accumulation in the snail hepatopancreas. Since dietary Cd fell below the detection limit, one can easily conclude that Cd transfer from soils to *C. aspersus* juveniles occurred independently of food ingestion. The soil ingestion and epithelial contact have been reported to be equally efficient in transferring soil Cd to land snails [13]. However, the present work cannot delineate between these routes of penetration because the test snails were exposed to Cd-contaminated soils as a single source of contamination.

The maximum hepatopancreas cadmium concentrations were above 500 mg/Kg d. wt. These values are among the highest Cd concentrations ever reported in the literature for this route of exposure. Because the internal Cd concentrations are not at equilibrium, land snails are expected to concentrate in the hepatopancreas higher amounts of Cd than those found in the present work. Indeed, such elevated accumulations have already been reported in the (eco)toxicological literature. For example, *Cepaea hortensis* showed hepatopancreas Cd levels above 1,600 mg/Kg d. wt when exposed for 14 days to dietary Cd doses of up to 254.14 mg/Kg d. wt [21].

The snails tended to concentrate Cd in the hepatopancreas relative to the soil, especially after 60 days of exposure to the highest Cd treatments. Most study authors reported lower levels of Cd in the viscera than in the soil, irrespective of exposure duration. The analyzed viscera included the digestive apparatus, kidney, heart, lung, and reproductive apparatus, and were routinely taken as a whole for assessing Cd bioaccumulation [8,13,15,18]. The hepatopancreas may therefore provide a more appropriate approach for characterizing the bioavailability of soil Cd than the use of snail viscera, as has already been proposed for biomonitoring metal accumulation in terrestrial ecosystems [45].

Cadmium addition to soils resulting from a new exposure event explained very well the dynamics of increase in Cd concentrations in the hepatopancreas. This implies that a new exposure event is an important factor contributing to the modification of Cd retention kinetics in the hepatopancreas of sub-adult *C. aspersus* previously exposed to Cd-spiked soils. Recently, we came to a similar conclusion regarding the soil manganese [26]. These data attest to the hepatopancreas of sub-adult *C. aspersus* the potential to serve as a sensitive bioaccumulator not only for soil Cd and Mn, but also for other metals that accumulate in soils.

The cumulative action of endogenous factors (i.e., age, body weight, physiological changes related to juvenile—adult transition) and exogenous factors (i.e., photoperiod, vapor pressure, temperature, mean rainfall level) accounted for 87% of the total Cd variance in soft tissue body burdens of *Cepaea nemoralis*. The age was the most influential factor and explained 80% of Cd variation in soft tissues [46]. The soil physicochemical properties, especially the pH and organic matter content, are known to regulate soil Cd bioavailailability to land snails [17]. As a result, future studies must be designed to elucidate more precisely the impact of a new exposure event on the kinetics of Cd accumulation in the hepatopancreas, for example, by implementing a mixed experimental design that allow scientists to distinguish between the overlapping effects of successive exposure events and by including all the aforementioned factors in a multiple regression model.

### Kinetics of cadmium in shell

Little knowledge exists on Cd accumulation in land snail shells for exposure via soil [8,16]. The current study fills this gap by demonstrating that soil Cd does accumulate in the shell independently of food ingestion. The measured values are among the highest ever recorded in the shell of terrestrial gastropods. Considerable Cd accumulations have also been reported in *Helix pomatia* that were sampled from two polluted areas in Romania, i.e., Medias and Copsa Mica. The average values were 5.99 and 3.72 mg/Kg d. wt Cd in the shell and 57.5 and 4.37 mg/Kg d. wt Cd in the soil, respectively [33]. Data in the literature generally show lower levels to which Cd accumulates in the gastropod shell. For example, the shell Cd content of *Cepaea nemoralis*, *Theba pisaba* and *Cepaea vindobonensis* respectively reached up to 0.36, 1.23, and 1.80 mg/Kg d. wt [35,36,47,48]. However, such comparisons must be treated with caution since even within closely related species of land snails there are significant differences in metal uptake and accumulation [49].

Food ingestion is more efficient in transferring Cd to land snails than exposure via soil [8]. Adult individuals of *C. aspersus* fed for 120 days on Cd-enriched diets (up to 125 mg/Kg d. wt) retained Cd in the shell at values up to 0.230 mg/Kg d. wt [32]. Not only different age, species and Cd bioavailability, but also methodological issues may contribute to explaining these differences. The snail shell consists mainly of calcium carbonate (CaCO_3_), but its outer layer, viz. the periostracum, contains a fibrous insoluble protein called conchiolin, which usually makes up to 5% of the shell [50–52]. This protein is an excellent biosorbent for metals, including Cd [51,52]. When directly treated with strong acids (e.g., nitric acid, hydrochloric acid), the shell is decalcified, while the acid-insoluble conchiolin precipitates [53]. Most of the aforementioned studies used strong acids to digest the dried shells without calcinating them prior to digestion [32,47,48]. This step produces an efficient destruction of the organic substances in the shell [54], and hence is expected to increase the concentration of Cd in the filtrates.

Cadmium accumulated to higher levels in the hepatopancreas than in the shell. Similar results were reported in previous studies [35,55]. The soil-to-organ regressions clearly indicate that the hepatopancreas is more sensitive than the shell in biomonitoring Cd accumulation in soils. Moreover, the shell Cd content remained well below 5% of that measured in either the soil or the hepatopancreas. Therefore, the shell cannot be regarded as a pertinent bioaccumulator for soil Cd, and environmental Cd broadly. This is consistent with the findings from studies on land snails and dietary Cd exposure [32,47].

There was no relationship between the increase in soil Cd content resulting from a new exposure event and the rate of Cd addition to the shell. This suggests that the first exposure event influenced more strongly the kinetics of Cd accumulation in the shell than a new exposure event. Terrestrial gastropods store calcium in the hepatopancreatic lime cells, whence it is later transported dissolved in the blood fluid into body parts in need of lime, especially towards the shell during the growth period [25]. Cadmium and calcium are competitive antagonists [56], and therefore, one potential explanation is that external Cd influx may reduce Ca transport from the hepatopancreas into the shell via competitive antagonism.

### Cadmium toxicity

Growth is a key ecophysiological factor for population sustainability. Cadmium toxicity has been assessed in terrestrial gastropods as a function of general growth inhibition [13,16], but never using only selected biometric features of the shell shape as sublethal end-points of exposure [8,37]. The temporal dynamics of shell height and shell width suggests here that the inhibition of shell growth occurs after relatively long exposure durations (60 days) to elevated soil Cd levels. Similar effects were reported for younger *C. aspersus* snails. Thus, one-month-old specimens were exposed to Cd-contaminated soils (up to 100 mg/Kg d. wt) under laboratory conditions; the minimal duration that significantly inhibited the growth of shell mass was 70 days [16].

The standard ISO-15952:2006 recommends the use of juvenile snails between three and five weeks old for assessing soil quality [22]. By contrast, the age of snails used in this study was three months. Not only the age-related decline in growth rate [25], but also environmental factors may account for our results, i.e., the reduced inhibitory effect of soil Cd on shell growth. Such factors (e.g., high temperatures, low relative humidity) are known to cause feeding cessation and sealing of shell aperture in land snails [25], thus limiting their growth. Laboratory data indicate that the body weight of sub-adult snails is a more sensitive end-point of exposure to soil Cd that the shell size/volume [27,37]. However, this parameter is highly variable in natural environments[27]. Therefore, both the shell size and body weight should be used with great caution in field research for assessing the sublethal effects of soil Cd.

Long-time exposure to metals has been linked to changes in shell size and allometry in adult land snails [1,48,57]. In our study, the shell robustness was not affected by soil Cd, but in adult *C. aspersus* it appeared to be influenced by long exposure to high Pb concentrations in the environment [39]. Hence, one can expect that significant changes in shell size and/or allometry may occur only in snail populations that are exposed to environmental Cd for several generations.

This study also provides scientists with pertinent information concerning the link between the hepatopancreatic Cd content and shell growth inhibition. Thus, under the present experimental conditions, the lowest-observable-effect concentration (LOEC) for the hepatopancreas cadmium should lie between 180 and 420 mg/Kg d. wt. Shell thinning and irregular growth around the aperture were the most frequently observed types of shell damage. Similar effects were reported at the growing edges of the common mussel (*Mytilus edulis*) when exposed to Cd-contaminated water [41], and as a consequence, we infer that Cd may interfere with Ca deposition in rapidly growing shell parts, that is around the shell aperture [25]. Such mechanisms are documented to exist in bones, wherein cadmium can antagonize and replace calcium in bone structure via competitive antagonism [58]. There is also indirect evidence that above a certain threshold level, Cd may inhibit Ca transport from the hepatopancreas into the shell. Thus, significant inhibition of shell growth and the most elevated frequencies of damaged shells were found for the highest Cd concentrations in the hepatopancreas.

Soil cadmium did not influence snail mortalities after 30 days of exposure, thus attesting to the high Cd tolerance of these invertebrates [16,32,37]. Not only Cd exposure, but also environmental factors appear to have contributed to high mortalities observed in the E2 phase since survival rates in control snails were significantly lower in this phase than in the E1 phase. This is most likely related to the fact that the peak of the summer 2011 temperatures in the study area was reached during the E2 phase [59]. Of note, in the E2 phase, mortality was higher for the second-highest-dose treatment than in snails exposed to the maximal soil Cd concentration. As a result, tolerance against Cd stress may increase with the level of the initial exposure dose in snails previously exposed to Cd-spiked soils.

A recent study has described dietary Cd accumulation in the hepatopancreas of *Helix pomatia* in relation with cadmium-induced metallothioneins (Cd-MT) synthesis of and snail mortality. The level at which the overburdening of Cd-MT with Cd^+2^ ions occurred was about 448 mg/Kg d. wt, and moreover, exposure above this level appeared to correlate with mortality [60]. Such information are not available for *C. aspersus*. In the present work, significant mortalities were observed in the E2 phase for hepatopancreas Cd concentration of 420 mg/Kg d. wt or more. One may assume that the Cd-MT overburdening threshold was exceeded during the E2 phase, but we infer that the observed mortalities may be better explained by the additive effect of environmental stress (mainly weather conditions) and progressive increase of Cd-MT saturation level with Cd exposure doses [60].

These results highlight the practical difficulties which arise when assessing cadmium toxicity using morphological end-points of exposure. In this context, more subtle biomarkers are required for assessing the potential hazard of Cd on land snails. Combining different histochemical, cytological, and cellular biomarkers of Cd exposure may represent a reliable option, as already described for *H. pomatia* [61]. Of particular interest is the potential use of epigenetic changes of Cd-MT gene promoter since there is strong indication for the existence of such a mechanism in land snails [62]. Such biomarkers have the potential to predict various adverse effects of Cd way before the first toxicity signs are observed at organismal, cellular, or subcellular level.

## Conclusions

This study shows, for the first time, that an increase in soil cadmium concentration resulting from a new exposure event changes the kinetics of cadmium retention in the hepatopancreas of snails previously exposed to cadmium-spiked soils. Both hepatopancreas and shell accumulated soil cadmium in a dose-dependent manner. However, the shell did not serve as a relevant bioaccumulator for this metal. Cadmium did not influence shell allometry, but at high levels significantly affected shell growth and snail survival, and also appeared to affect shell integrity in rapidly growing shell parts. Our results attest to the value of hepatopancreas for describing cadmium retention in land snails and to the difficulty of using conchological parameters in field surveys for estimating the environmental hazard of soil cadmium.
